# Theranostics in Sarcoma: A Critical Review of Current Evidence and Future Potential

**DOI:** 10.1007/s11864-026-01386-0

**Published:** 2026-03-26

**Authors:** Ojasvi Appana, David Konieczkowski, Gabriel Tinoco

**Affiliations:** 1 College of Medicine, The Ohio State University, Columbus, OH 43235 USA; 2 Department of Radiation Oncology, The Ohio State University, Columbus, OH 43235 USA; 3 Division of Oncology, Department of Internal Medicine, The Ohio State University Comprehensive Cancer Center, Columbus, OH 43235 USA

**Keywords:** Theranostics, Sarcoma, Review, Soft-tissue sarcoma, Molecular imaging, Personalized medicine

## Abstract

Sarcomas are rare, heterogeneous malignancies with over 100 subtypes and limited effective therapies in advanced stages. Theranostics, a strategy that combines diagnostic imaging and targeted radionuclide therapy using the same molecular target, has revolutionized care in cancers, including prostate and neuroendocrine tumors. This review evaluates the current evidence and future potential of theranostic applications in sarcoma. A comprehensive literature search was conducted across PubMed, Google Scholar, and clinicaltrials.gov for studies published between January 2010 and August 2025. Search terms included “theranostics,” “radionuclide,” “molecular imaging,” “sarcoma,” and molecular targets such as PSMA, FAPI, SSTR, PDGFR, and TEM-1. Eligible articles included clinical trials, cohort studies, and systematic reviews. Case reports were included only when higher-level evidence was lacking. Key theranostic targets in sarcoma include FAP, PSMA, SSTR, and PDGFR. Among these, FAP-directed agents demonstrate the most clinical maturity, with improved diagnostic performance compared to 18F-FDG and early evidence of disease control with 90Y/177Lu-labeled therapies. PSMA and SSTR expression in sarcomas is more variable and localized to tumor vasculature, with case-level therapeutic use and limited efficacy data. PDGFR and emerging targets, such as TEM-1, show biological promise but remain under investigation in early-phase studies. Theranostics represents a promising approach to individualized sarcoma treatment, with FAP-targeted agents showing the most advanced clinical development. FAP-based imaging improves lesion detection compared to standard 18F-FDG PET, and early therapeutic trials suggest disease control in select patients. However, broader validation and regulatory approval are needed before clinical integration. Theranostics represents a promising precision-medicine paradigm for sarcoma, linking diagnostic PET imaging with targeted radioligand therapy targeting a shared molecular target. With more than 100 histologic subtypes and limited effective treatment options in advanced disease, theranostics may provide a future therapeutic avenue for selected patients. Among currently investigated platforms, fibroblast activation protein-directed approaches are the most clinically advanced. 68Ga FAPI PET has demonstrated superior lesion detection compared with 18 F FDG across multiple sarcoma subtypes, and early studies of 90Y- and 177Lu-labeled FAPI agents report disease stabilization in selected patients with advanced disease, supporting biological and technical feasibility. In contemporary practice, FAP-directed imaging is best integrated within investigational frameworks to identify candidates for radioligand therapy, refine trial selection, and enable response monitoring in FAP-avid sarcomas. Other targets, including PSMA, SSTR, and PDGFR, remain exploratory and require prospective validation before clinical translation. Standard sarcoma management continues to rely on guideline-directed multimodality care, including surgery, radiation, and subtype-specific systemic therapy, with theranostic strategies currently limited to clinical trials or selected refractory settings. Broader clinical adoption will depend on prospective studies demonstrating meaningful clinical benefit, optimized dosimetry, and regulatory approval.

## Introduction

Sarcomas are rare and diverse tumors, accounting for only 1% of adult malignancies yet consisting of over 100 different histological and molecular subtypes [1]. The subtypes are broadly classified into 2 main groups: bone sarcoma (BS) and soft-tissue sarcoma (STS). With their complex biology and diverse clinical characteristics, sarcomas pose significant challenges for diagnosis and treatment. Current treatment strategies include surgery, often in combination with adjuvant chemotherapy or radiation. However, particularly in the recurrent or metastatic setting, sarcomas are often resistant to these treatments and frequently have a poor prognosis [1–3]. Recently, treatment guidelines have shifted from a one-size-fits-all approach toward a more personalized approach tailored to histological subtype and clinical presentation [1, 4]. Despite these advances, there remains a need for innovative and effective therapeutic options. Theranostics, a dual-purpose strategy that integrates diagnostics and therapy on a single platform, has the potential to advance the emerging precision medicine paradigm in sarcoma.

Theranostics combines target-specific positron emission tomography (PET) or single-photon emission computed tomography (SPECT) imaging with a matched radioligand therapy (RLT), enabling a unified approach to patient selection, individualized dosing through image-based dosimetry, and real-time monitoring of treatment response, all within the same molecular pathway [5–7]. Subsequently, a therapeutic agent is administered that targets the same or a closely related molecular structure identified during imaging. This approach enables real-time, non-invasive assessment of target expression, facilitating both accurate patient selection and ongoing evaluation of treatment effectiveness. Importantly, by visualizing the molecular target prior to therapy, theranostics addresses the challenge of spatial and temporal heterogeneity in target expression, which is particularly relevant in sarcoma and other solid tumors [6].

While the concept of theranostics dates back over 70 years, with the introduction of radioactive iodine in the diagnosis and treatment of thyroid cancer, its use in other cancers is relatively new. Regulatory approvals for [^177^Lu]Lu-dotatate in neuroendocrine tumors (NETs) and for [^177^Lu]Lu-vipivotide tetraxetanin prostate cancer illustrate mature diagnostic-therapy pairing in other cancers; however, no such approvals exist for sarcoma [5–7]. This review aims to summarize current evidence, identify promising molecular targets, and evaluate the clinical relevance and limitations of theranostic strategies for sarcoma.

Importantly, across all theranostic strategies evaluated to date in sarcoma, available therapeutic evidence remains limited to small, non-randomized cohorts and compassionate-use experiences. No studies have yet demonstrated survival benefit, durable tumor control, or improvement in patient-reported outcomes. Accordingly, reported responses should be interpreted as proof of biological and technical feasibility rather than confirmation of clinical efficacy. At present, no theranostic approach can be considered part of standard sarcoma management, and all radioligand therapies discussed in this review should be regarded as investigational and restricted to research settings.

**Fig. 1:**
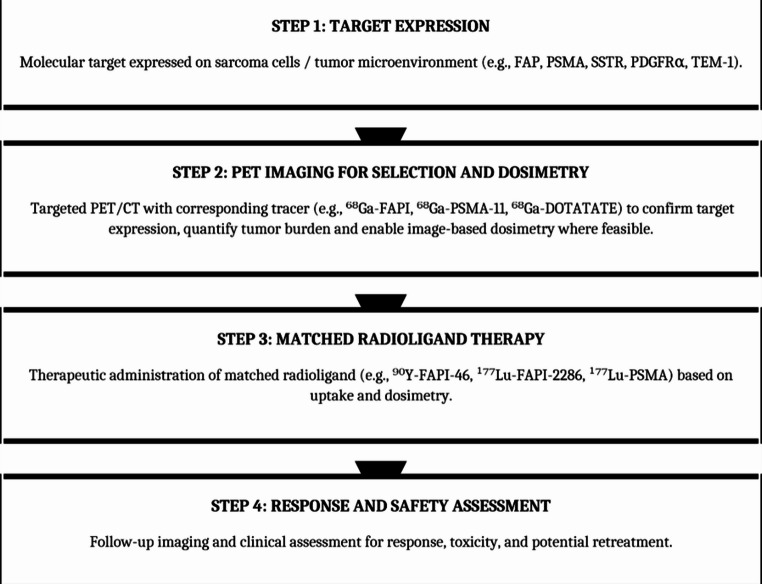
Theranostic workflow in sarcoma. The theranostic paradigm integrates diagnostic imaging and targeted therapy using the same molecular pathway. Step 1: A molecular target is identified on sarcoma cells or within the tumor microenvironment. Step 2: Targeted PET/CT imaging with a radiolabeled tracer confirms target expression, quantifies tumor burden, and enables image-based dosimetry to predict therapeutic radiation dose delivery. insufficient uptake or unfavorable dosimetry may preclude therapy. Step 3: A matched radioligand bearing a therapeutic beta-emitting isotope (⁹⁰Y or ¹⁷⁷Lu) is administered, targeting the same molecular pathway identified during imaging. Step 4: Follow-up imaging and clinical assessment evaluate treatment response, monitor for toxicity, and guide decisions regarding additional therapy cycles. Abbreviations: FAP, fibroblast activation protein; PSMA, prostate-specific membrane antigen; SSTR, somatostatin receptor; PDGFRα, platelet-derived growth factor receptor alpha; TEM-1, tumor endothelial marker 1; PET/CT, positron emission tomography/computed tomography

The sequential steps required for theranostic translation, from target identification to matched radioligand therapy and response assessment, are summarized in Fig. 1.

## Methods

### Study Design and Objectives

This work was conducted as a narrative, target-focused review aimed at summarizing current and emerging applications of theranostic imaging and radionuclide therapy in sarcoma, organized by molecular target rather than by study design or hierarchical level of evidence. The primary objective was to describe available clinical and translational data for each target and to identify gaps that may inform future research priorities and clinical trial design.

### Eligibility Criteria

Reports were eligible for inclusion if they met all of the following criteria:

1. Included patients with soft-tissue or bone sarcoma, with sarcoma-specific data extractable when mixed tumor cohorts were reported;
2. Evaluated a radiolabeled probe or therapeutic agent directed against a defined molecular target (for example, fibroblast activation protein, PSMA, somatostatin receptors, PDGFR, or TEM-1) for diagnostic imaging, radionuclide therapy, or both; and
3. Provided original clinical, translational, or imaging data from prospective or retrospective studies, clinical trials, observational cohorts, or case series.

Studies were excluded if they consisted solely of preclinical experiments without human data, involved non-sarcoma cohorts from which sarcoma-specific results could not be extracted, or focused exclusively on technical aspects of physics, radiochemistry, or dosimetry without associated human imaging or treatment outcomes. Single-patient case reports were included only when higher-level evidence was unavailable for a given target or sarcoma subtype and were clearly identified as such in the text and tables.

### Information Sources and Search Dates

A targeted literature search was performed in PubMed/MEDLINE and Google Scholar to identify relevant clinical and translational studies. An initial search was conducted between 1 May 2024 and 30 August 2024, followed by an updated search on 30 August 2025 to capture more recent publications and newly registered studies. ClinicalTrials.gov was queried over the same period to identify ongoing or completed trials evaluating theranostic imaging or radionuclide therapy in sarcoma, using sarcoma-related terms and target-specific keywords. Reference lists of key articles and reviews were manually screened to identify additional relevant studies not retrieved through database searches.

### Search Strategy

**Fig. 2:**
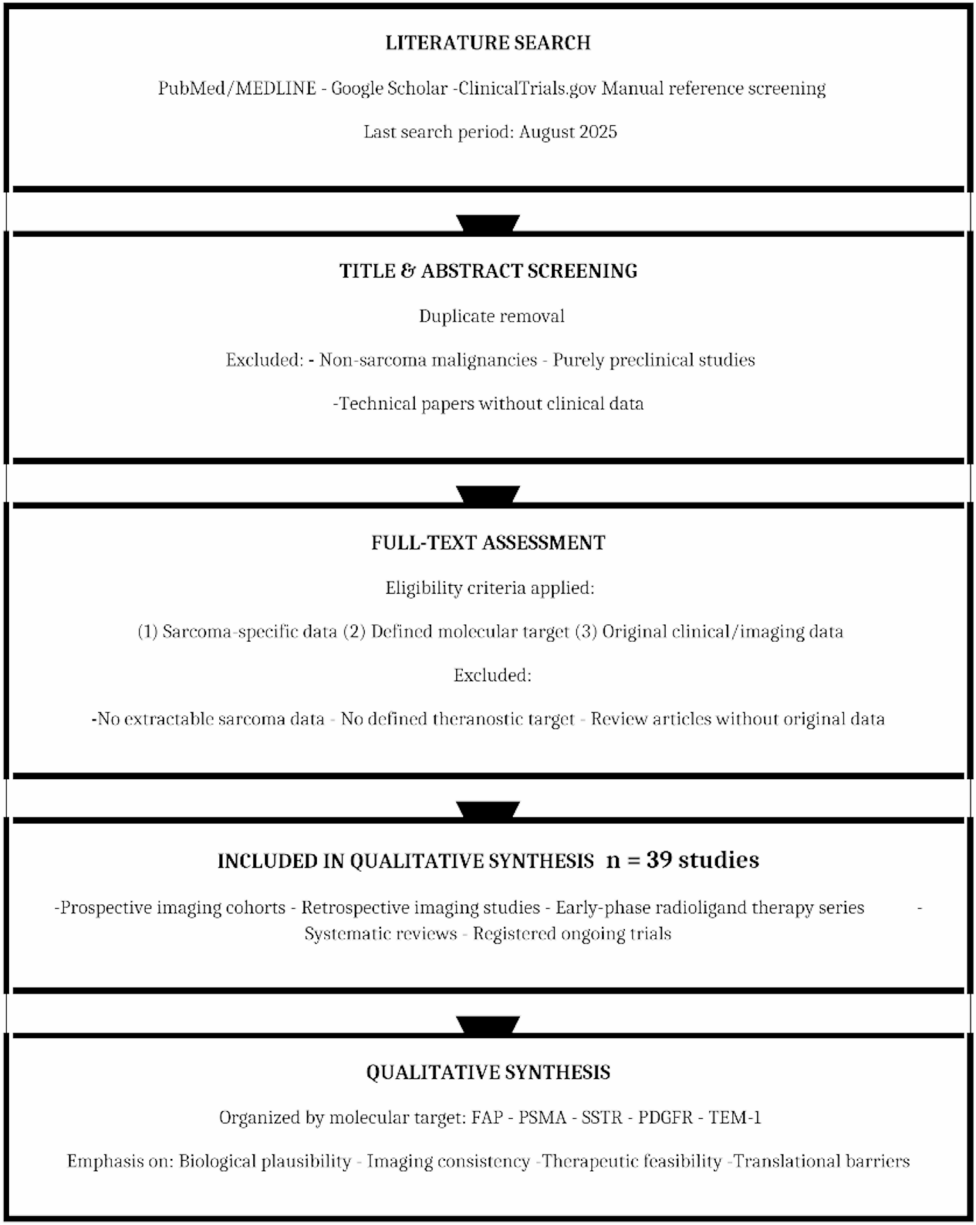
Study selection flow for narrative review of theranostics in sarcoma. This narrative, target-focused review did not employ formal PRISMA methodology or independent duplicate screening. The search across PubMed/MEDLINE, Google Scholar, ClinicalTrials.gov, and manual reference screening yielded 39 eligible studies organized by molecular target for qualitative synthesis. Studies included prospective and retrospective imaging cohorts, early-phase radioligand therapy series, systematic reviews, and registered ongoing trials. No automation tools or quantitative pooling methods were used. Data were synthesized qualitatively with emphasis on biological plausibility, consistency of imaging findings, feasibility of therapeutic delivery, and identification of translational barriers relevant to future clinical development

Because this was a narrative, target-focused review, search strategies were adapted for each molecular target rather than constructed as a single comprehensive systematic search string. For PubMed/MEDLINE, combinations of controlled vocabulary and free-text terms were used, including “sarcoma”, “soft tissue sarcoma”, “bone sarcoma”, “osteosarcoma”, and “liposarcoma”, combined with target-specific terms such as “fibroblast activation protein”, “FAP”, “PSMA”, “prostate specific membrane antigen”, “somatostatin receptor”, “SSTR”, “PDGFR”, “platelet derived growth factor receptor”, and “TEM-1”, together with modality-related terms including “PET”, “positron emission tomography”, “radioligand therapy”, “radionuclide therapy”, and “theranostic”. No language restrictions were applied at the search stage, although only articles with an English abstract were considered for full-text review. Filters for human studies were applied where available, and no date limits were imposed beyond the final search date. Similar keyword combinations were used in Google Scholar and ClinicalTrials.gov, with manual review of the initial result sets to identify relevant records (Fig. 2).

### Study Selection

Titles and abstracts retrieved from all sources were screened to remove duplicates and exclude clearly irrelevant reports, including non-sarcoma malignancies, purely preclinical studies without human data, and technical physics or radiochemistry papers without associated human imaging or treatment outcomes. Full texts were obtained for the remaining records and assessed against the predefined eligibility criteria. Screening was performed by one author, with a second author independently reviewing inclusion decisions in cases of uncertainty; disagreements were resolved by discussion and consensus. Given the narrative scope of the review and the limited size of the evidence base for many targets, formal independent duplicate screening and a PRISMA-style flow diagram were not implemented. No automation tools were used at any stage of the screening or selection process. The search and screening process yielded 39 eligible studies for inclusion in the qualitative synthesis, comprising prospective and retrospective imaging cohorts, early-phase radioligand therapy series, systematic reviews, and registered ongoing trials.

### Data Extraction and Synthesis

From each eligible study, data were collected using structured summaries organized by molecular target. Extracted variables included study design; sample size and sarcoma subtypes; molecular target and radiolabeled agent; imaging modality; therapeutic agent and administered activity, where applicable; key imaging findings; reported treatment responses; safety signals; and any dosimetry or pharmacokinetic information relevant to theranostic application. Data extraction was performed by one author and independently checked for accuracy and completeness by at least one additional author during preparation of the final tables and narrative summaries, with discrepancies resolved by consensus.

Formal risk-of-bias assessment, effect-size calculation, subgroup analysis, sensitivity analysis, or quantitative pooling was not performed, as the available literature consisted predominantly of small, heterogeneous cohorts and early-phase studies not suitable for statistical synthesis. Instead, findings were synthesized qualitatively within each target domain, with emphasis on biological plausibility, consistency of imaging signal, feasibility of therapeutic delivery, and translational barriers relevant to future clinical development.

## Results

Key theranostic targets that have been explored for use in sarcoma include FAP, PSMA, SSTR, and PDGFR. (Clinical studies evaluating theranostic imaging and radioligand therapy in sarcoma are summarized in Table 1).

**Table 1:** Clinical studies evaluating theranostic imaging and RLT in sarcoma

Author	NCT number	Target	Theranostic agent(s)	Study type		Number of patients	Design / population	Key diagnostic findings	Key therapeutic findings	Safety summary
Kessler et al., [12]	NCT04571086	FAP	^68^Ga-FAPI	Diagnostic only	47		Prospective observational trial in patients with BS and STS	^68^Ga-FAPI PET/CT showed superior lesion detection and uptake versus ^18^F-FDG, particularly in low-grade and intermediate-risk sarcomas.	Not applicable.	No significant tracer-related adverse events reported.
Lanzafame et al., [13]	NA	FAP	^68^Ga-FAPI	Diagnostic only	200		Prospective cohort of BS and STS	^68^Ga-FAPI PET/CT improved detection of intermediate and low-grade sarcomas and identified candidates for FAPI RLT.	Not applicable.	No grade 3–4 toxicities reported.
Kleiburg et al., [17]	NCT05522257	PSMA	^18^F-JK-PSMA-7	Diagnostic only	25		Prospective single-center study in adults with metastatic STS	High PSMA uptake observed in a subset of patients, but heterogeneous tracer distribution across lesions limited suitability for PSMA-targeted therapy.	Not applicable.	No significant adverse events reported.
Tinoco et al., [28]	NCT06500065	SSTR	^68^Ga-DOTATATE	Diagnostic only	Planned n (STS)		Ongoing prospective trial in STS	Study ongoing; designed to evaluate diagnostic performance and SSTR expression in STS.	Not applicable.	Not yet reported.
Telix Pharmaceuticals [33]	NCT06537596	PDGFR	^89^Zr-olaratumab	Diagnostic only	Planned n (STS)		Prospective study in STS	Ongoing; evaluates radiolabeled olaratumab PET for PDGFR targeting, biodistribution, and dosimetry.	Not applicable.	Not yet reported.
Fendler et al., [15]	NA	FAP	^90^Y-FAPI-46 ± prior ^68^Ga-FAPI	Diagnostic + therapeutic	21 (16 with metastatic sarcoma)		Single-center retrospective cohort including patients with metastatic sarcoma and other solid tumors	FAPI PET demonstrated high target uptake and informed RLT eligibility and dosimetry.	In sarcoma, a subset achieved metabolic and/or radiologic disease control; no survival benefit established.	Grade 3–4 hematologic toxicity (thrombocytopenia, anemia) was the main adverse event.
Ferdinandus et al., [34]	NA	FAP	^90^Y-FAPI-46	Diagnostic + therapeutic	9 (6 with STS/BS)		Retrospective monocentric study of advanced solid tumors, including STS/BS	Baseline FAPI PET confirmed target expression and permitted individualized dosimetry.	Radiologic and metabolic disease control in a proportion of sarcoma patients; responses generally short to intermediate.	Hematologic adverse events, including grade 3 thrombocytopenia; no acute severe reactions.
Banihashemian et al., [35]	NCT05522257	FAP	^177^Lu-FAPI-2286	Diagnostic + therapeutic	8 (all metastatic sarcoma)		Prospective pilot study in metastatic sarcoma	Baseline FAPI PET identified FAPI-avid lesions as RLT targets.	Significant reduction in lesion size and disease control in several evaluable patients; no survival advantage yet demonstrated.	No grade 3–4 toxicity observed.
Prior et al., [36]	NCT05420727	PSMA	^68^Ga-PSMA-11 / ^177^Lu-ITG-PSMA-1	Diagnostic + therapeutic	Planned n (STS)		Prospective feasibility trial in STS	Designed to assess PSMA PET/CT for target expression and eligibility for PSMA RLT; results pending.	Therapeutic outcomes not yet reported.	Not yet reported.

Abbreviations: STS, soft-tissue sarcoma; BS, bone sarcoma; PET, positron emission tomography; RLT, radioligand therapy. “Study type” indicates whether the report was limited to diagnostic imaging (“diagnostic only”) or included both imaging and therapeutic radionuclide administration (“diagnostic + therapeutic”). “Number of patients” reflects the sarcoma subset within mixed-tumor cohorts where applicable

FAP, a transmembrane serine protease, is a particularly useful target because it is overexpressed on the surfaces of cancer-associated fibroblasts (CAFs) as well as sarcoma cells. Its endopeptidase activity enables the synthesis of FAPI that serve as molecular probes [8]. FAPI ligands can be labeled for imaging with 68Ga or ^18^F and for therapy with ^177^Lu or ^90^Y, leveraging FAP’s high expression on CAFs and reported expression on sarcoma cells [8–10].

FAPI PET has rapidly progressed into prospective clinical evaluations across tumor types, supporting its translation to sarcoma cohorts [11]. In prospective cohorts, 68Ga-FAPI PET improved lesion detection and diagnostic accuracy versus ^18^F-FDG, identifying potential candidates for FAP-radiopharmaceutical therapy [12, 13].

Early FAP-RLT tests, using ^90^Y-FAPI-46 and ^177^Lu-FAPI-2286, report metabolic or radiologic disease control in sarcoma subtypes (mainly solitary fibrous tumors in this study), with cytopenia as the principal observed toxicity [14, 15]. These findings support biological and technical feasibility but do not establish clinical benefit. Prospective, controlled trials demonstrating durable and patient-relevant benefit are required before FAP-RLT can be considered beyond investigational use.

A recent systematic review by Abdulrahman et al., [16] evaluated the diagnostic and theranostic role of FAPI PET/CT in soft tissue sarcoma, synthesizing 36 clinical studies comprising 316 patients across approximately 30 histologic subtypes. That analysis demonstrated consistently high FAPI uptake, improved lesion detection compared with ^18^F‑FDG PET/CT, and the feasibility of FAPI‑based radioligand therapy, with disease stabilization reported in a substantial proportion of treated patients and an overall favorable safety profile. Within this broader landscape, current evidence suggests that FAP‑directed approaches represent the most clinically mature theranostic pathway in sarcoma, whereas PSMA‑ and SSTR‑based strategies remain limited by heterogeneous expression and sparse therapeutic data, and PDGFR‑ and TEM‑1–directed platforms remain largely investigational. Across targets, imaging‑based patient selection, standardized uptake thresholds, and dosimetry‑driven treatment protocols emerge as critical prerequisites for future clinical translation.

Overall, FAP represents the most clinically mature theranostic pathway currently under investigation in sarcoma. The evidence base includes multiple prospective imaging cohorts, consistent correlation between tracer uptake and histopathologic FAP expression, and early-phase radioligand therapy series reporting disease control in selected patients. From a readiness perspective, FAPI PET is technically robust and increasingly available, and patient selection for therapy using uptake thresholds is feasible in principle, although no FAP-directed radioligand therapy is approved for sarcoma. Key limitations remain substantial, including small, predominantly single-center cohorts; heterogeneity in uptake across subtypes and lesions; variability among FAPI derivatives; and incomplete dosimetry reporting, all of which constrain interpretation of efficacy and hinder standardization.

Another key theranostic target is PSMA, a type II transmembrane glycoprotein, which, despite the name, is expressed in a variety of tumors, including some sarcomas [17]. PSMA tracers labeled with ^68^Ga or ^18^F for diagnostic purposes, or with ^177^Lu for therapeutic ones, have become standard in patients with prostate cancer. In sarcoma, PSMA localizes predominantly to tumor neovasculature rather than tumor cell membranes (as in prostate cancer), and higher expression has been associated with adverse features [17, 18]. Expression is correlated with tumor size, metastasis risk, and overall worse clinical outcome, making it a potential prognostic marker [8]. Studies of PSMA expression across sarcoma grades and subtypes have shown variable expression, with the strongest levels observed in high-grade tumors and in the neovasculature of pleomorphic rhabdomyosarcomas and synovial sarcomas .

Case-level evidence shows that ^68^Ga-PSMA-11 and ^68^Ga -PSMA PET/CT can potentially detect disease in certain sarcoma subtypes (leiomyosarcoma, osteosarcoma, and liposarcoma) although uptake is heterogeneous within and across lesions [19–22]. Therapeutic experience with ^177^Lu-PSMA and ^177^Lu-PSMA-617 in sarcoma remains limited to single-patient reports with variable benefit, underscoring the need for prospective selection by imaging and dosimetry [21, 22]. Prospective studies aim to further characterize PSMA expression across histological sarcoma subtypes and evaluate the efficacy of ^68^Ga -PSMA PET/CT and ^177^Lu-PSMA RLT [23, 24].

In sarcoma, the level of evidence supporting PSMA-targeted theranostics remains limited, consisting largely of expression studies, small prospective imaging cohorts, and isolated therapeutic case reports. Imaging demonstrates variable uptake that is frequently confined to tumor neovasculature, which complicates both lesion detection and reliable patient selection for therapy. From a clinical readiness standpoint, this pattern of expression limits confidence in target engagement and dosimetry-based treatment planning, rendering therapeutic translation premature. Major limitations include pronounced inter- and intra-patient heterogeneity, absence of defined uptake thresholds predictive of response, and lack of prospective radioligand therapy trials, all of which currently restrict PSMA-directed strategies to exploratory investigation in sarcoma.

A third potential target is SSTR 2 (SSTR2), an endogenous signaling molecule that is overexpressed in many cancers, including most STSs [8]. SSTR-targeted theranostics are well-established in NETs, but their prevalence and utility in sarcoma remain uncertain, pending dedicated studies [25]. One theranostic agent, ^68^Ga-DOTATOC, is known to be sensitive to and specific for meningiomas and has aided in the differential diagnosis of synovial sarcoma; however, its utility in sarcoma-specific imaging remains uncertain [26]. Another published case study utilized the theranostic agent Lu-DOTA0-Tyr3-octreotate as a therapeutic agent for synovial sarcoma, with results showing potential efficacy and tolerability. However, further studies are needed to characterize its effectiveness [27]. An ongoing trial (NCT06500065) is testing the diagnostic efficacy of ^68^Ga-DOTATATE in STS [28].

Evidence supporting SSTR-directed theranostics in sarcoma is sparse and derives almost entirely from isolated diagnostic observations and rare therapeutic case reports. In contrast to neuroendocrine tumors, systematic data on receptor prevalence, uptake distribution, and correlations with clinical outcomes are lacking. As a result, clinical readiness remains low, with no validated framework for patient selection or for expectations regarding therapeutic benefit. Key limitations include the uncertain frequency of expression across sarcoma subtypes, the absence of comparative imaging studies, and the lack of prospective trials evaluating radioligand therapy, placing SSTR-targeted approaches in sarcoma firmly within a hypothesis-generating domain.

PDGFR is another potential sarcoma theranostic target. Expression of PDGF ligands and receptors varies across sarcoma histologies, with PDGFA and PDGFRα signaling particularly relevant in synovial sarcoma [32]. Preclinical inhibition of PDGFRα can attenuate growth and metastatic behavior, although translation to clinical benefit has proven challenging [30]. Despite biologic plausibility, the ANNOUNCE phase 3 trial showed no survival benefit for doxorubicin plus olaratumab over doxorubicin alone, emphasizing the need for imaging-based enrichment and patient selection in future studies [31]. Immuno-PET with ^89^Zr-olaratumab is under prospective evaluation to confirm in vivo PDGFRα engagement and define dosimetry in STS (ZOLAR, NCT06537596) [29].

Although PDGFR signaling is biologically relevant across several sarcoma subtypes, current evidence supporting theranostic applications is limited to preclinical data and early translational imaging studies. No clinical experience with radioligand therapy in sarcoma has been reported, and imaging strategies remain investigational, primarily limited to immuno-PET platforms. Consequently, clinical readiness is low, with no established role for PDGFR-directed imaging in treatment selection or response assessment. Major limitations include a lack of human therapeutic data, uncertainty regarding target accessibility for radionuclide delivery, and prior failures of PDGFR-targeted systemic therapies, all of which temper expectations for near-term theranostic translation.

## Discussion

Theranostics has become a critical tool in the management of some cancers, including NETs and meningiomas (for example, with SSTR-targeted approaches) and prostate cancer (with PSMA-targeted approaches). Building on this success, and the diagnostic and therapeutic difficulties posed by sarcoma, the application of this paradigm to STS is promising.

Of the theranostic pathways under investigation in sarcoma, FAP currently has the most mature human evidence: prospective imaging studies demonstrate higher lesion detection than ^18^F-FDG and identify candidates for RLT, while early FAP-RLT cases show disease control in subsets with expected hematologic toxicity, supporting dose optimization and phase 2 validation. By contrast, PSMA expression in sarcoma reflects predominantly neovasculature involvement, with heterogeneous PET uptake and only case-level therapeutic experience; therefore, future PSMA-RLT trials should pair quantitative PET with tissue-level profiling and dosimetry. SSTR-targeted approaches remain hypothesis-generating outside of neuroendocrine tumors (NETs), with isolated diagnostic and therapeutic anecdotes; prevalence studies and the determination of standardized imaging thresholds are required [12, 15, 18, 21, 24–26, 27, 34, 37]. Notably, lutetium Lu-177 dotatate and lutetium Lu-177 vipivotide tetraxetan are United States Food and Drug Administration (FDA)-approved for NETs and prostate cancer, respectively, but not for sarcoma; sarcoma-specific trials are needed before their use can be considered for sarcoma [38, 39].

Limitations across the literature include small, single-center cohorts; a lack of randomized controls; variable uptake thresholds; and incomplete dosimetry reporting – all of which can inflate apparent effect sizes and complicate cross-study comparisons [15].

Future directions for sarcoma theranostics will require a multifaceted effort. First, a more comprehensive survey of theranostic targets across sarcoma subtypes is needed. If target expression still varies significantly within each subtype, an efficient method will be required to identify patient-specific target expression. On that basis, larger controlled trials will be needed to determine the diagnostic performance and therapeutic efficacy of theranostic agents; collaborative networks and multicenter trials will be essential. Critical to this effort will be matched pairs of diagnostic/therapeutic agents, enabling paired PET imaging and RLT. In addition to the targets described in this review, new theranostic targets are needed, particularly in aggressive sarcoma subtypes that do not respond well to existing treatments. One potential new target is TEM, otherwise known as endosialin. TEM-1, a transmembrane glycoprotein, is overexpressed in tumors like sarcoma on mesenchymal stromal fibroblasts and pericytes [40]. One study evaluating TEM-1 expression revealed expression across all 19 sarcoma subtypes tested [41]. Thus far, preclinical studies have shown promise in the efficacy of the radio-iodinated MORAb-004 antibody, which can detect human TEM-1, and anti-TEM1Fc with saporin, which can kill sarcoma cells in vivo [41, 42]. Clinical studies will be required to determine its true value as a theranostic target in practice.

## Conclusion

Among currently investigated targets, FAP-directed approaches have advanced furthest along the translational pathway, supported by prospective imaging cohorts and early therapeutic feasibility studies, whereas other strategies remain limited by heterogeneous target expression, sparse clinical evaluation, or predominantly preclinical development. Prospective ^68^Ga-FAPI PET improves lesion detection compared with ^18^F-FDG and enables imaging-based identification of candidates for radioligand therapy, while early ^90^Y/^177^Lu-FAPI studies report disease control in selected patients with cytopenias as the principal observed toxicity. PSMA-targeted approaches predominantly reflect neovasculature-associated uptake and are supported by case-level therapeutic experience only; further progress will require quantitative PET-based selection, tissue-level validation, and systematic dosimetry. SSTR-directed strategies remain exploratory in sarcoma outside of established indications such as neuroendocrine tumors and meningioma. None of these radioligand therapies is approved for use in sarcoma.

Next steps are therefore pragmatic and testable. Trials should incorporate imaging-enriched designs with predefined uptake thresholds, require organ and marrow dosimetry, harmonize PET-derived metrics with anatomic response criteria, and evaluate rational combination strategies within dose-constrained frameworks. Whether theranostics can deliver durable and patient-relevant benefit across biologically diverse sarcoma subtypes will depend on the rigorous application of these principles.

## Key References

• Lanzafame H, et al. 68Ga Fibroblast Activation Protein Inhibitor PET CT Improves Detection of Intermediate and Low Grade Sarcomas and Identifies Candidates for Radiopharmaceutical Therapy. J Nucl Med. 2024;65:880–87.
  - ◦ This reference is of outstanding importance because it reports the largest prospective sarcoma cohort to date (*n* = 200) demonstrating superior lesion detection with 68Ga FAPI PET compared with 18 F FDG across histologic subtypes and direct identification of patients suitable for radioligand therapy, establishing the most robust clinical imaging evidence supporting FAP directed theranostics in sarcoma.
• Abdulrahman M, et al. Exploring the Diagnostic and Theranostic Role of FAPI PET in Soft Tissue Sarcoma A Systematic Review. Nucl Med Mol Imaging. 2025;59:289–305.
  - ◦ This reference is of outstanding importance because it synthesizes 36 clinical studies comprising 316 patients and confirms consistently high FAPI uptake, improved lesion detection compared with FDG, and early feasibility of radioligand therapy, providing the most comprehensive evaluation of the diagnostic and translational role of FAP based theranostics in sarcoma.
• Banihashemian SS, et al. Feasibility of 177Lu FAPI 2286 Theranostics in Patients with Advanced Metastatic Sarcoma. Eur J Nucl Med Mol Imaging. 2024;52:237–46.
  - ◦ This reference is of outstanding importance because it demonstrates clinical feasibility of 177Lu FAPI radioligand therapy in metastatic sarcoma, showing measurable lesion reduction, disease control in selected patients, and manageable toxicity, representing one of the earliest prospective signals supporting therapeutic application of FAP directed theranostics.
• Tinoco G. 68Ga DOTATATE PET CT for the Diagnosis of Soft Tissue Sarcomas. NCT06500065. 2025. Available from ClinicalTrials.gov.
  - ◦ This reference is of importance because it represents the first prospective study specifically evaluating somatostatin receptor imaging in soft tissue sarcoma, addressing a critical evidence gap and expanding theranostic investigation beyond FAP toward alternative molecular targets.

## Data Availability

No new data were created or analyzed in this study.

## Declarations

### Use of Generative AI and AI-Assisted Technologies in the Manuscript Preparation Process

During the preparation of this work, the author(s) used Grammarly in order to improve orthography and readability. After using this tool/service, the author(s) reviewed and edited the content as needed and take(s) full responsibility for the content of the published article.
